# The emerging roles of exosomal circRNAs in diseases

**DOI:** 10.1007/s12094-020-02485-6

**Published:** 2020-09-15

**Authors:** X. Guo, W. Tan, C. Wang

**Affiliations:** 1 School of Medicine, South China University of Technology, Guangzhou, 510006 People’s Republic of China; 2 Guangdong Provincial People’s Hospital, Guangdong Geriatric Institute, Guangdong Academy of Medical Sciences, Guangzhou, 510080 People’s Republic of China

**Keywords:** Exosomes, circRNAs, Exosomal circRNAs, Function, Liquid biomarker

## Abstract

Exosomes, the nanoscale phospholipid bilayer vesicles, enriched in selected proteins, nucleic acids and lipids, which they participated in a variety of biological processes in the body, including physiology and pathology. CircRNAs (circular RNAs) are a class of single-stranded closed molecules with tissue development specific expression patterns that have crucial regulatory functions in various diseases. Non-coding RNAs (such as microRNAs and long non‑coding RNAs) in exosomes have also been shown to play an important regulatory role in humans. However, little research has focused on exosomal circRNAs. Recently, CircRNAs have been identified to be enriched and stably expressed in exosomes. In this review, we summarize the biogenesis and biological functions of exosomes and circRNA, and further revealed the potential role of exosome-derived circRNA in different diseases. Besides, we propose its use as a diagnostic marker and therapeutic punctuation for diseases, especially in cancer.

## Introduction

Exosomes, 30–150 nm in size, originate from the membrane vesicles of the endosomes and are secreted by almost all cells from different organisms, and are considered to be key roles in biological processes under normal and pathological conditions [1, 2]. Exosomes contain a variety of functional molecules, including various growth factors, proteins, metabolites, DNA, lipids, transfer RNA (tRNA), messenger RNA (mRNA), ribosomal RNA (rRNA), microRNA (miRNA), long non‑coding RNA (lncRNA), circRNA and so on [3]. Numerous studies have demonstrated that exosomes are crucial mediators of intercellular communication. After exosomes are released into the tissue fluid, through a series of movements, exosomes arrive at the recipient cells and begin to deliver their internal active substances (such as circRNAs), thus initiating functional responses and inducing subsequent phenotypic changes [4–6].

Exosomes not only target the target cells to trigger downstream signals but also transfer genetic material to target cells, exerting anti-inflammatory, antiapoptotic and immune-suppressive effects, promoting tissue repair and improving cytokine levels [7, 8]. In addition, exosomes are involved in the development of many pathological processes such as in cancer, diseases of the nervous system and endocrine system [9–11]. Exosomes accumulate and circulate freely in different body fluids, and also contain a variety of functional molecular carriers. Therefore, exosomes may become a very promising non-invasive liquid biomarker, which will play an important role in future clinical diagnosis and treatment [12, 13]. CircRNAs are new endogenous non-coding RNAs, which are produced by splicing events or back-splicing events via exons or (and) introns circularization of the original transcription [14]. With the classical linear RNA with 5′caps and 3′poly(A) tails, circRNAs have a covalent closed-loop structure to avoid being degraded by RNA external enzymes or RNase R, so the number of circRNAs is more stable than linear mRNA, which makes them more rich than the typical linear transcription products of the same gene [15]. Due to the application of RNA sequencing (RNA-seq) technology coupled with novel bioinformatics approaches, circRNA has been found to occur widely and stably in human, animal and plant cells, even in mammalian tissues [16]. CircRNAs could regulate gene expression at transcriptional, posttranscriptional and translational levels. They participate in many pathological processes such as Alzheimer’s disease, diabetes, atherosclerosis and myogenesis through regulating alternative splicing, sponging miRNAs, sequestering functional proteins or even encoding proteins [17–20]. Particularly, circRNAs play an important role in cancer growth, metastasis, recurrence and therapy resistance [21]. Surprisingly, recent studies have shown that large and stable expression of circRNA can be detected in exosomes extracted from human circulation and urine [22]. For instance, it has been reported that tumor-derived exosomes are involved in the development, invasion and metastasis of gastric cancer, and circRNA can be transmitted to recipient cells through exosomes to promote the malignant process of gastric cancer [23, 24]. Moreover, exosomal circRNAs were also found in pancreatic ductal adenocarcinoma (PDAR) [25], hepatocellular carcinoma [26], platelet-derived extracellular vesicles [27]. As can be seen from the above studies, exosome circRNAs may participate in a variety of life activities and pathological processes in the human body, and the combination of the two may further increase the potential clinical applications of these molecules as diagnostic and prognostic markers. In the past, exosomal lncRNAs and miRNAs have been widely reported, but relatively little attention has been devoted to exosomal circRNAs. In this review, we briefly address the biogenesis and biological functions of exosome and circRNAs, the significance and role of exosomal circRNAs in various diseases. Besides, we propose exosomal circRNAs can as a promising diagnostic marker and therapeutic punctuation for diseases, especially in cancer.

## Biogenesis and function of exosome

**Fig. 1:**
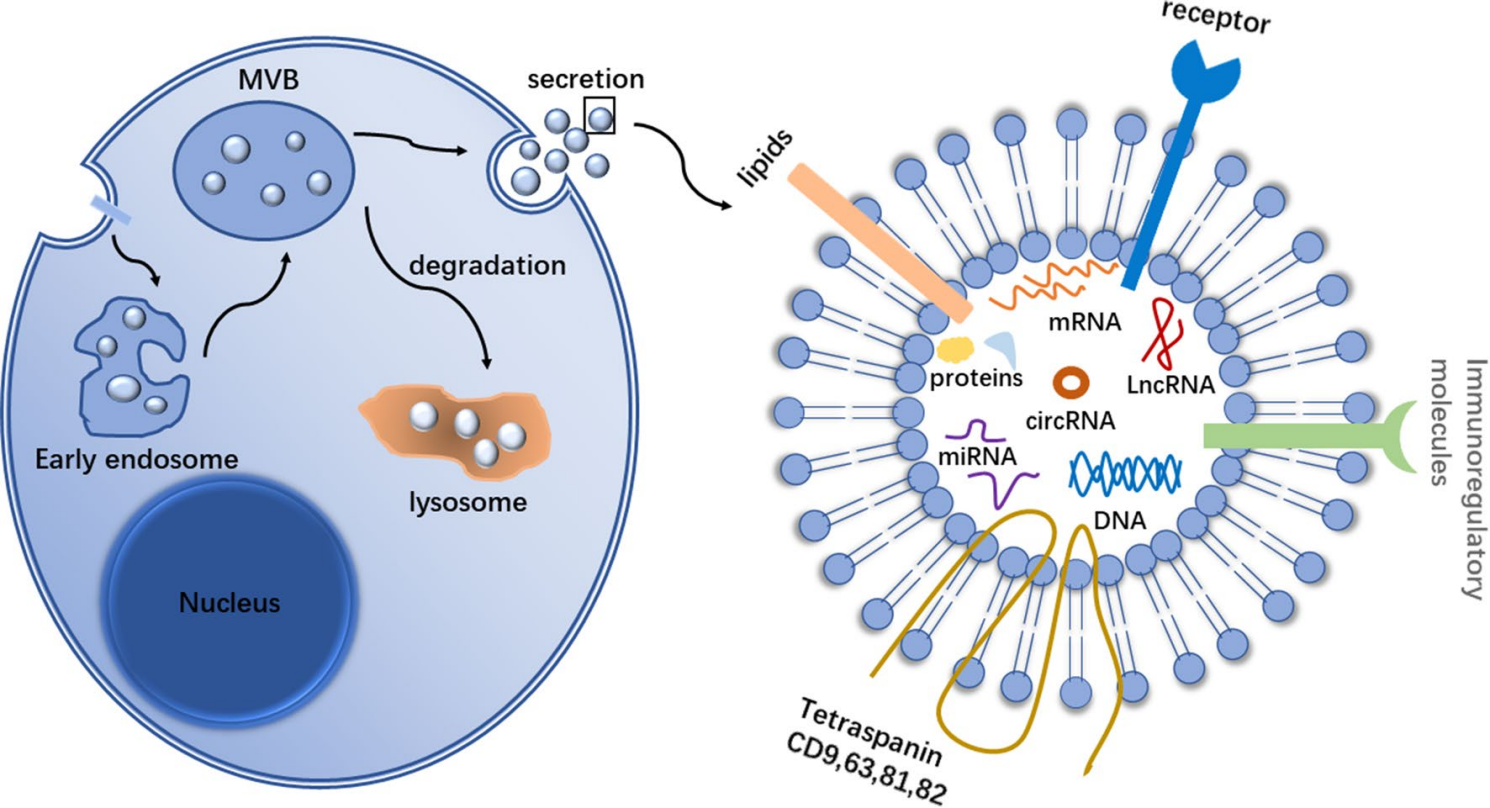
Biogenesis and structure of exosome

Exosomes are a type of extracellular vesicle consisting of a phospholipid bimolecular cell membrane, which are found in various body fluids such as blood, saliva, urine, breast milk and so on [28]. Early endosomes were formed by intracavity vesicles on the plasma membrane, which were further folded by the phospholipid bilayer to produce multiple intracavity vesicles, thus forming multi-vesicular body (MVB). It could not only fuse with the plasma membrane to release its contents, namely exosomes, but also transport to lysosomes to degrade the contents of vesicles [29, 30]. In addition, the membranes of exosomes are rich in a variety of proteins and lipids, such as cholesterol and sphingolipids. There are other proteins on the surface of exosomes, including four major transmembrane proteins (CD9, CD63, CD81 and CD82), which are enriched on their membranes and considered as ideal markers for exosome characterization [31], as shown in Fig. 1. It is noteworthy that exosomes play an important role in regulating normal physiological processes, such as repair ischemic injury [32], healthy pregnancy [33] and tissue repair [34]. In addition, exosomes also participate in the development of various diseases in the human body, playing a role that cannot be ignored. Studies have shown that CD317 protein and epidermal growth factor receptor (EGFR) are highly concentrated on the surface of exosomes in serum of non-small cell lung cancer (NSCLC), which is therefore considered as an important marker for the diagnosis of NSCLC [35]. Exosomes released by cancer cells can mediate phenotypical changes in the cells of tumor microenvironment (TME) to promote tumor growth and therapy resistance, for example, fibroblast- and macrophages-induced differentiation [36]. Therefore, exosomes are biomarkers with diagnostic and prognostic significance, and we will further study their potential in clinical application.

Early endosomes were formed by intracavity vesicles on the plasma membrane, which were further folded by the phospholipid bilayer to produce multiple intracavity vesicles, thus forming MVB. It can fuse with the plasma membrane to release its contents, namely exosomes, also transport to lysosomes to degrade the contents of vesicles.

The structure of exosomes include, tetraspanins (CD9, CD63, CD81, CD82). Lipid rafts, such as sphingolipids, cholesterol. Immunoregulatory molecules include major histocompatibility complex I, II (MHC I, MHC II), CD86. The receptors on the exosome membrane are different depending on the host cell, eg. EGFR. Exosome contents have proteins, nucleic acids such as DNA, tRNA, mRNA, rRNA, miRNA, lncRNA, circRNA and so on.

## Isolation and detection of exosomes

Exosomes have great potential in clinical diagnosis, biomarker detection, target recognition of molecular therapy and personalized medical application. However, there are still some obstacles such as super-sensitive detection technology, efficient isolation method and quantitative analysis. In the clinical environment, a fast and reliable test standard is urgently needed. A large number of approaches have been used for exosome and extracellular vesicle (EV) isolation, such as ultracentrifugation, density-gradient centrifugation, size exclusion chromatography, microfiltration, immunomagnetic beads, and several polymer-based precipitation techniques [37–41]. These separation techniques rely either on the size and density of the EVs or the existing specific surface proteins on the exosomes such as, CD9, CD63, CD81, TSG101 and HSP70 [41]. Fortunately, with the in-depth research, different approaches with recognition element were reported, including nanoparticles tracking analysis (NTA), tunable resistive pulse sensing (RPS), dynamic light scattering (DLS), flow cytometry, cryo-EM, a platform combining surface plasmon resonance (SPR) with atomic Force Microscope (AFM), or by other techniques with similar capabilities have been developed [42–44]. Besides, Western Blotting can be utilized to verify the presence of biomarker proteins in EVs. Traditional flow cytometry is suitable for identification of large vesicles, but not nanoscale ones. High-sensitivity flow cytometer (HSFCM) has been found that can increase the detection limit from about 500 nm to as low as 40 nm, making it possible to detect other large and small vesicle populations, such as exosomes [45]. Although these techniques and methods are fast-changing, there are still many limitations and shortcomings in their clinical application. First, many of methods require state-of-the-art instruments. Second, impure recoveries with regard to remnant matrix species (e.g., proteins, genetic material) and are performed on clinically irrelevant time and volume scales. Third, the selectivity for some specific exosomes is deficient. Fourth, the accuracy is often influenced by unreliable nanoparticle counting, which is from large proteins and lipoprotein particles, and even some exosomes less than a certain size being omitted [46]. We expect that in the future a technology will be developed to break down these barriers and translate exon detection of disease sources into clinical applications for early screening in primary care settings.

## Origins, biogenesis and functions of circRNA

### Origins of circRNA

CircRNA was discovered as early as the 1970s, in 1976, Sanger et al. obtained a source of viroids from tomatoes and purified the viroid RNA. Hydrodynamic and thermo-dynamic studies proved that circRNA exists in the viroid [47], which is an earlier research study we discovered that proved the existence of circRNA. But CircRNAs were thought to be a result of splicing errors for several decades, so scientists have paid little attention to how the molecule works in humans [15]. Subsequently, circRNAs have since been found in humans, mice, rats, fungi and other organisms [48–50]. However, with the development of modern technology, many advanced methods for circRNA detection and identification have been developed, such as bioinformatics and high-throughput sequencing technologies, which provide further convenience for us to study the biological functions of circRNA. Since then, circRNA-related research has grown rapidly and has gradually become a new star in the non-coding endogenous RNA field.

### Biogenesis of circRNA

**Fig. 2:**
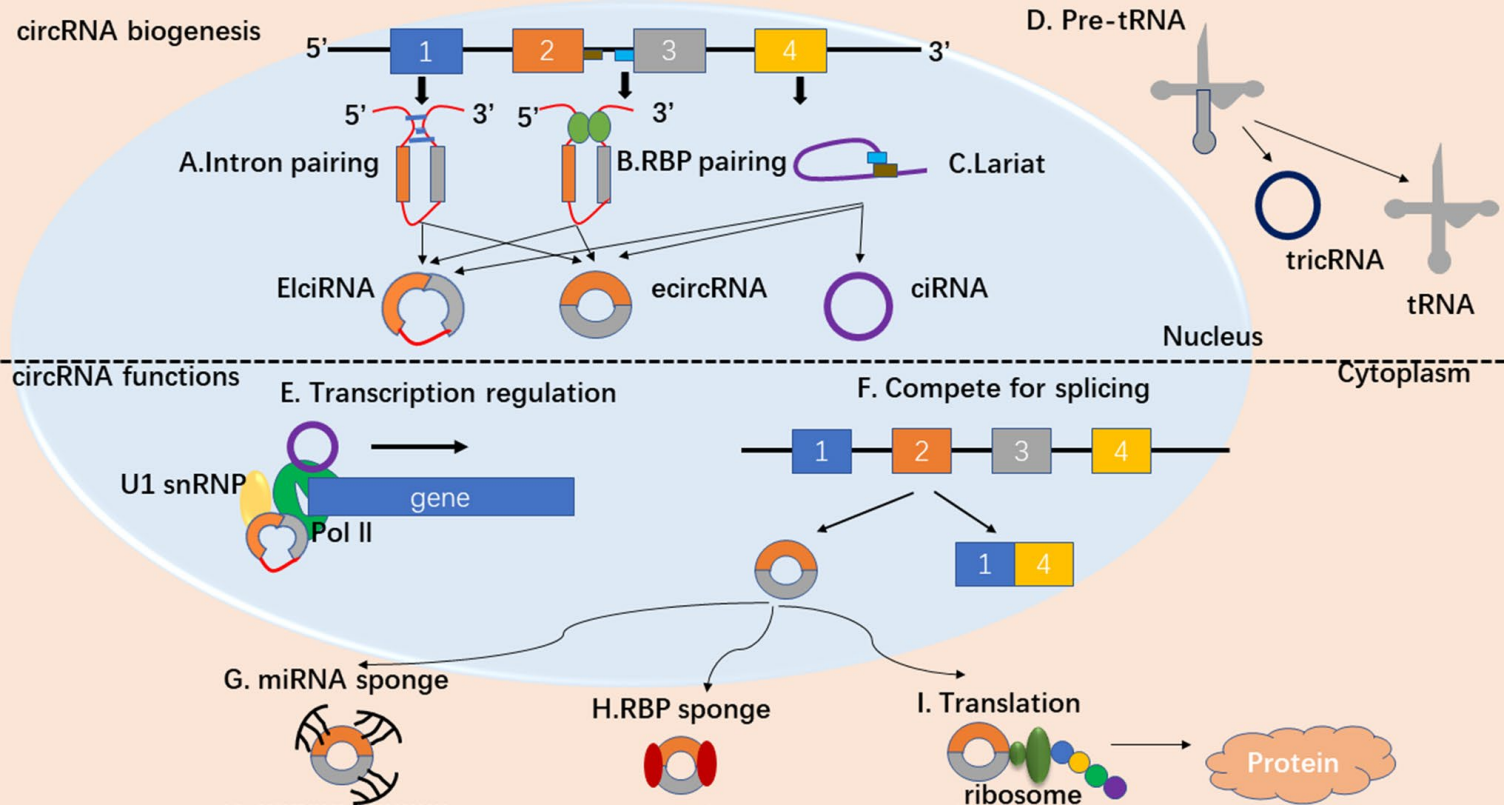
Biogenesis and function of circRNAs. **a** Intron pairing driven circularization. The cycle driven by intron pairing is realized by direct base pairing of introns on both sides of complementary sequences or reverse repeats. The introns are removed or retained to form ecircRNA or EIciRNA. **b** RBP pairing driven circularization. The RBPs are embedded into the precursor RNA during transcription and bind to the lateral intron sequence, causing the precursor RNA to bend and fold to form a circular cross region. **c** Lariat-driven circularization. It takes place in exon jump events or the removal of introns from the precursor mRNAs. **d** TricRNAs are synthesized from introns spliced from pre-tRNA. The tRNA splicing endonuclease complex cuts an intron-containing pre-tRNA at a canonical bulge-helix bulge (BHB) motif; and the intron termini are also joined by RtcB ligase to form a stable circRNA, named tricRNA. **e** EIciRNAs or ciRNAs can interact with transcription complexes in the promoter region of their host gene to induce gene transcription by interacting with U1 snRNP or RNA pol II. **f** The back-splicing and linear splicing can compete with each other during splicing. As a result, a linear RNA or an ecircRNA is generated. **g** circRNAs can act as miRNA sponges to inhibit miRNA activity. **h** circRNAs can interact with RBPs and affect their functions and translocations. **i** circRNAs have protein-coding capacity and can encode proteins

circRNAs differ from other RNAs in their remarkable continuous closed-loop structure, which is covalently linked by free 3′and 5′ends [51]. This closed-loop structure, which is also called a “back-splicing” structure, which require the spliceosome machinery to join a 5′ splice site (donor) of an exon to an 3′ splice site (acceptor) [52]. The biogenesis of circRNAs as shown in Fig. 2, which can be divided into three types: ciRNAs (circular intron RNAs), Ecircnas (circular exon RNAs), and EIciRNAs (exon–intron circular RNAs) [53]. The formation mechanism is as follows: the cycle driven by intron pairing is realized by direct base pairing of introns on both sides of complementary sequences or reverse repeats [54, 55]. In the RBP (RNA-associated binding protein) associated pairing drive, the RBPs are embedded into the precursor RNA during transcription and bind to the lateral intron sequence, causing the precursor RNA to bend and fold to form a circular cross region [56]. RBP pairing and intron pairing drive circularization in the direct back-splicing pathway of circRNA formation. In lariat-driven models, it takes place in exon jump events or the removal of introns from the precursor mRNAs [57]. In addition, another conserved mode of circRNA biogenesis is a small part of intron-derived circRNAs are from pre-tRNA. During pre-tRNA maturation, the tRNA splicing endonuclease complex cuts an intron-containing pre-tRNA at a canonical bulge-helix bulge (BHB) motif; and the intron termini are also joined by RtcB ligase to form a stable circRNA, named tricRNA [58, 59], as shown in Fig. 2.

### Functions of circRNA

CircRNAs are new molecules in the field of ncRNA research, and their biological functions have been extensively studied. Their functions can be divided into four parts: as miRNA sponges regulating their functions, as transcription or translation regulators, interacting with proteins to regulate gene expression, and having the potential to encode proteins. These functions are shown in Fig. 2.

#### miRNA sponge

CircRNAs have been shown to act as miRNA sponges that can isolate miRNAs from their target mRNAs, thereby alleviating miRNA-mediated gene suppression or expression. The most typical example is Cdr1as, abundant circRNAs in mammalian brain, which contains more than 70 conserved miR7 binding sites and miR-671 sites that are completely complementary to the entire mature miR-671 sequence and can directly regulate the function of miRNAs and their collaborative gene [60]. CircHIPK3 has been shown to promote cancer cell growth by binding to multiple miRNAs, including the tumor suppressor miR-124 [61]. The existing results significantly demonstrate that the circRNA-miRNA interaction is critical for the organism.

#### Regulation of transcription and alternative splicing

Although circRNAs are mostly exons and distributed in the cytoplasm, ciRNA and EIciRNA are abundant in the nucleus and play a role as transcriptional regulatory cis-elements [62]. Some circRNAs regulate the transcription of their parent genes by promoting the extended activity of RNA polymerase II (Pol II) [62, 63]. EIciRNA with Pol II and its coding gene U1 small nuclear ribonucleoprotein (U1 snRNP) promoter interaction, enhance its parent gene transcription [64]. Therefore, although ciRNA and EIciRNA cannot function as miRNA sponges, they can regulate gene expression and transcription through other pathways, which has been confirmed in studies. Back-splicing can also compete with linear mRNA splicing, so disruption of the balance between circular and linear RNA may promote abnormal gene expression, which may be further involved in pathological activity [56].

#### Translation

Some circRNAs have an open reading frame (ORF) and an internal ribosomal binding site (IRES), showing potential for protein translation [65–67]. CircLINC-PINT can encode the peptide chain and inhibit the transcription of oncogenes, thus playing an anticancer role in glioblastoma [68]. Another study showed that in the presence of translation initiation factors EIF4G2 and YTHDF3, modification of N6-methyladenosine (m6A) was sufficient to promote the initiation of circRNAs protein translation in human cells [69].

#### Interactions with RBPs

CircRNAs may have the potential to regulate protein function by binding, storing, sequencing and isolating proteins to specific subcellular locations, and act as dynamic scaffold molecules regulating protein–protein interactions [70]. Recent studies have shown that eight RNA-binding proteins interact with circRNAs in liver cancer cells, and SCD-circRNA 2 can regulate RNA-binding sequence protein 3 (RBM3) and play an important role in liver cancer [71]. On the other hand, circRNAs can influence the translation process by competitively binding RBP to their mRNAs,and the extensive binding of circPABPN1 to HuR prevented the interaction between HuR and PABPN1, thus inhibiting the translation of PABPN1 [72]. All the evidence suggests that circRNAs are more likely to regulate protein function by acting as scaffolds for protein complexes rather than simply binding to individual proteins.

## Origin and mechanism of exosomal circRNAs

Numerous studies have shown that exosomes contain various nucleic acid substances, such as mRNA, miRNA, lncRNA, rRNA, circRNA, etc., when they transferred to the recipient cells, causing a series of biological reactions and play an important role in regulating the physiological process of cells [73–75]. Moreover, the rich and stable expression of circRNAs in extracellular vesicles has been identified, providing new clues and support for the functional research of circRNAs. It was shown that compared with parental cells, circRNAs in exosomes were more abundant, larger in number and more stable in expression than linear mRNA subtypes [22, 76]. Possible mechanisms for circRNAs to be more abundant in exosomes than in its producing cells include, first, the relatively long half-life of circRNAs, as they are covalently closed-loop structures without a poly A tail or a 5′–3′ end, thereby avoiding degradation by endonuclease [62]. Studies have shown that most circRNAs have a half-life of more than 48 h, while linear RNAs have a half-life of less than 20 h. Therefore, compared with linear transcripts in cells, circRNAs are more stable and enriched [15]. Second, the extracellular vesicles release circRNAs, which are then exported to the extracellular microenvironment to form a mechanism for circRNA clearance, so circRNAs are more enriched in the extracellular space [77]. Finally, due to the protective effect or certain characteristics of the exosome envelope, it can safely prevent further removal of contents or degradation by RNA enzyme, so exosomal circRNAs are highly stable and abundant [78]. Exosomes contain different types of active substances whose sources reflect the state and type of cells; however, exosomes from different or identical sources may contain the same or different molecules, indicating that the molecular subsets in exosomes are tissue-specific and uniquely species-specific. Reverse transcriptional quantitative PCR and high-throughput analysis were used to detect exosome-derived circRNA, and the results revealed the difference of circRNA types in serum and exosomes [22], which also indicated that circRNA entered exosomes actively, including selectivity in the sorting process [79]. In addition, studies have shown that RNA-binding complexes endosoma-sorting complex required for transport II (ESCRT‑II) play an important role in the development of MVB and may help EV to selectively incorporate RNA [1]. Other studies have shown that intracellular miRNA may regulate the process of circRNA entering exosomes [22].

In summary, we need to further investigate and elucidate the complex mechanisms by which RNAs selectively and specifically enters exosomes, and we speculate that multiple types of sorting mechanisms may be at work simultaneously. These findings will greatly expand the potential of circRNAs as a prognostic and diagnostic biomarker.

## Exosomal circRNAs in cancers

Increasing evidences show that exosomal circRNAs play a crucial role in a variety of cancers and participate in many important biological processes, promoting or inhibiting cancer. It could provide us with further insights into the functional mechanism of exosomal circRNAs. Table 1 illustrates the role of some exosomal circRNAs in different cancers.

**Table 1:** Dysregulation of exosomal circRNAs in cancer biology

circRNA	Cancer type	Expression	Biological function	Mechanism	Citation
circ-0051443	HCC	Downregulated	Promote cell apoptosis and arrest the cell cycle	circ-0051443/miR-331-3p/BAK1	[80]
circ-DB	HCC	Upregulated	Promote growth and reduce DNA damage	circ-DB/miR-34a /USP7	[81]
Cdr1as	HCC	Upregulated	Promote proliferation and migration	Cdr1as/miR-1270/AFP	[82]
circNRIP1	GC	Upregulated	Promote tumour metastasis	circNRIP1/miR-149-5p/AKT1	[83]
ciRS-133	GC	Upregulated	Promote cancer-associated cachexia	ciRS-133/miR-133/PRDM16	[5]
Circ-RanGAP1	GC	Upregulated	Promote invasion and metastasis	circ-RanGAP1/miR-877-3p/VEGFA	[24]
circLONP2	CRC	Upregulated	Promote invasion and metastasis	circLONP2 /miR-17/DGCR8	[84]
ciRS-122	CRC	Upregulated	Promote glycolysis and drug resistance	ciRS-122/miR-122/PKM2	[85]
circ-ABCC1	CRC	Upregulated	Promote cell stemness, sphere formation and metastasis	circ-ABCC1/Wnt /β-catenin	[86]
circIFT80	CRC	Upregulated	Promote the proliferation, migration and invasion	circIFT80 /miR-1236-3p /HOXB7	[87]
circFMN2	CRC	Upregulated	Promote proliferation and migration	circFMN2/miR-1182 /hTERT	[88]
circ-PDE8A	PDAC	Upregulated	Promote proliferation and invasion	Circ-PDE8A /miR-338/MACC1	[25]
Circ-IARS	PDAC	Upregulated	Promoting tumor invasion and metastasis	circ-IARS/miR-122/RhoA	[89]
CircPUM1	ovarian cancer	Upregulated	Promote proliferation, migration and invasion	CircPUM1 /miR-615-5p/miR-6753-5p/NF-Κb/MMP2	[90]
CircWHSC1	ovarian cancer	Upregulated	Promote proliferation and metastasis	CircWHSC1/miR-145/miR-1182	[91]
Cdr1as	ovarian cancer	Downregulated	Inhibit proliferation and promot cisplatin-induced cell apoptosis	Cdr1as/miR-1270/SCAI	[92]
CircSATB2	NSCLC	Upregulated	Promotes proliferation, migration and invasion	circSATB2/miR-326/FSCN1	[93]
Circ_0044516	prostate cancer	Upregulated	Promote growth and metastasis	Circ_0044516/miR‐29a‐3p	[94]
CircPRMT5	UCB	Upregulated	Promote growth and metastasis	circPRMT5/miR-30c/SNAIL1/E-cadherin	[95]
circRASSF2	LSCC	Upregulated	Promote proliferation	circRASSF2/miR-302b-3p/IGF-1R	[96]
mc-COX2	CLL	Upregulated	Promote proliferation and inhibit apoptosis	–	[97]
CircRASSF2	PTC	Upregulated	Promote proliferation and inhibit apoptosis	CircRASSF2/miR-1178/TLR4	[98]

*HCC* hepatocellular carcinoma, *GC* gastric cancer, *CRC* colorectal cancer, *PDAC* pancreatic ductal adenocarcinoma, *NSCLC* non-small cell lung cancer, *UCB* urothelial carcinoma of the bladder, *PTC* papillary thyroid carcinoma, *LSCC* laryngeal squamous cell carcinoma, *CLL* chronic lymphocytic leukemia

### Hepatocellular carcinoma (HCC)

Circ-0051443 is significantly lower in the plasma exosomes and tissues from patients with HCC than healthy controls. Circ-0051443 is transmitted from normal cells to HCC cells via exosomes and suppresses the malignant biological behaviors by promoting cell apoptosis and arresting the cell cycle. Therefore, exosomal circ-0051443 can serve as a predictor and potential therapeutic target for HCC [80].

exosome circ-deubiquitination (circ-DB) is up-regulated in HCC patients with higher body fat ratios. Moreover, exo-circ-DB promotes HCC growth and reduces DNA damage via the suppression of miR-34a and the activation of deubiquitination-related USP7 [81]. Exosomes extracted from HCC cells overexpressing circRNA Cdr1as accelerated the proliferative and migratory abilities of surrounding normal cells. In all, circRNA Cdr1as serves as a ceRNA to promote the progression of HCC. Meanwhile, it is directly transferred from HCC cells to surrounding normal cells exosomes to further mediate the biological functions of surrounding cells [82]. The study aimed to identify differentially-expressed circRNAs (DECs) in human blood exosomes from patients with HCC and to investigate their diagnostic value. Additionally, compared with the normal samples, hsa_circ_0004001, hsa_circ_0004123, hsa_circ_0075792, and a combination of the three were utilized as valuable diagnostic biomarkers in HCC [99].

### Gastric cancer (GC)

It was proven that circNRIP1 can be transmitted by exosomal communication between GC cells, and exosomal circNRIP1 promoted tumor metastasis in vivo. In addition, circNRIP1 sponges miR-149-5p to affect the expression level of AKT1 and eventually acts as a tumor promotor in GC [83]. Exosomes derived from GC cells deliver ciRS-133 into preadipocytes, promoting the differentiation of preadipocytes into brown-like cells by activating PRDM16 and suppressing miR-133. Thus, exosome-delivered circRNAs are involved in white adipose tissue (WAT) browning and play a key role in cancer-associated cachexia [5]. Circ-RanGAP1 was significantly up-regulated in both GC tissues and exosomes from the plasma of GC patients. High circ-RanGAP1 expression was closely associated with an advanced TNM stage, lymph node metastases, and worse survival. The circ-RanGAP1-mediated miR-877-3p/VEGFA axis promotes GC progression [24]. Low hsa_circ_0065149 expression levels in GC tissues were significantly associated with tumor diameter, perineural invasion and longer overall survival. More important, hsa_circ_0065149 levels were significantly decreased in plasma exosomes of early GC patients. As a screening biomarker for early GC, hsa_circ_0065149 in plasma exosomes has higher sensitivity and specificity than traditional clinical biomarkers [100].

### Colorectal cancer (CRC)

Studies indicate that circLONP2 acts as key metastasis-initiating molecule during CRC progression through modulating the intracellular maturation and intercellular transfer of miR-17, resulting in dissemination of metastasis-initiating ability in primary site and acceleration of metastasis formation in foreign organs. circLONP2 could serve as an effective prognostic predictor and/or novel anti-metastasis therapeutic target in CRC treatment [84]. Furthermore, exosomes from oxaliplatin-resistant cells delivered ciRS-122 to sensitive cells, thereby promoting glycolysis and drug resistance through miR-122 sponging and PKM2 upregulation [85]. Exosomes from CD133 cells carrying circ-ABCC1 can promote cell stemness, sphere formation and metastasis in CRC, unveiling that circ-ABCC1 serves as a novel candidate target for CRC treatment [86]. Studies have shown that hsa_circ_0067835 (circIFT80) and hsa-circ-0005100 (circFMN2) were significantly up-regulated in exosomes from CRC patients’ serum. They could promote the proliferation, migration and invasion of cancer cells. These results showed that these exosomal levels of circRNAs were tumor-derived and they can serve as a diagnostic or prognostic biomarker for CRC [87, 88]. Cancer-derived exosomal circPACRGL plays an oncogenic role in CRC proliferation and metastasis. CircPACRGL serves as a sponge for miR-142-3p/miR-506-3p to facilitate the transforming growth factor-β1 (TGF-β1) expression, providing mechanistic insights into the roles of circRNAs in CRC progression and a valuable marker for CRC treatment [101].

### Pancreatic cancer

Exosomal circ-PDE8A was associated with progression and prognosis in PDAC patients. Circ-PDE8A acts as a ceRNA for miR-338 to regulate MACC1 and stimulates invasive growth via the MACC/MET/ERK or AKT pathways [25]. Circ-IARS was up-regulated in plasma exosomes in pancreatic cancer tissues and patients with metastatic disease, and was found to enter HUVECs through exosomes, promoting tumor invasion and metastasis. In addition, circ-IARS expression was positively correlated with liver metastasis, vascular infiltration and tumor-node-metastasis (TNM) stage, and negatively correlated with postoperative survival time, suggesting that circ-IARS may be a potential prognostic marker for pancreatic cancer [89].

### Ovarian cancer

CircPUM1 up-regulated the expression of nuclear factor kappa B (NF-κB) and MMP2 by sponging miR-615-5p and miR-6753-5p to promote ovarian cancer proliferation, migration and invasion, which also acts on the peritoneum and contributes to metastasis of cancer in the form of cancer-derived exosomes [90]. CircWHSC1, which is highly expressed in ovarian cancer, acts on the peritoneal mesothelium in the form of exosomes to induce tumor metastasis. It is also found to promote tumor development through sponging miR-145 and miR-1182 [91]. Cdr1as was down-regulated in serum exosomes from cisplatin-resistant patients. Overexpression of Cdr1as inhibited cell proliferation and promoted the cisplatin-induced cell apoptosis in ovarian cancer cells by regulating the miR-1270/SCAI signaling pathway [92].

### Breast cancer

Both of hsa-circRNA-00005795 and hsa-circRNA-0088088 may function as competing endogenous RNAs and may play vital roles in BCa, which hold significant value for the prediction of BCa by examining the expression of the circRNAs in serum exosomes from BCa patients compared with that in healthy donors. The study provides a theoretical basis for using stable exosomal circRNAs as new biomarkers for predicting the tumorigenesis, development and metastasis of BCa [102].

### Lung cancer

The CXCR4-related circular RNA exo-hsa_circRNA_0056616 in exosomes was more expressed in lung adenocarcinoma tissues with lymph node metastasis. This circRNA represents a potential biomarker for predicting lymph node metastasis in lung adenocarcinoma [103]. CircSATB2 is highly expressed in serum exosomes of lung cancer patients, with high sensitivity and specificity in clinical detection, and is associated with lung cancer metastasis. In addition, circSATB2 promotes proliferation, migration and invasion of NSCLC cells through exosome. The results revealed that circSATB2 may be a promising diagnostic marker in lung cancer [93]. CircRNA-002178 could be delivered into CD8 T cells to induce PD1 expression via exosomes. It could be detected in exosomes of plasma from lung adenocarcinoma (LUAD) patients and could serve as biomarkers for LUAD early diagnosis [104].

### Urinary system cancer

Circ_0044516 was verified to be significantly up-regulated in the exosomes from prostate cancer patients and the cell lines. Inhibition of circ_0044516 expression in prostate cancer cells decreased prostate cancer cell growth and metastasis, which regulated prostate cancer cell biological functions by sponging miR‐29a‐3p [94]. CircPRMT5 was up-regulated in serum and urine exosomes from patients with UCB, and significantly correlated with tumor metastasis. Clinical analysis from two independent UCB cohorts showed that the circPRMT5/miR-30c/SNAIL1/E-cadherin pathway was essential in supporting UCB progression [95].

### Other tumors

There is a circRASSF2/miR-302b-3p/insulin-like growth factor 1 receptor (IGF-1R) axis in LSCC progression. Importantly, study demonstrated that circRASSF2 was up-regulated in serum exosomes from LSCC patients, indicating the significance of exosomal circRNAs in tumor cell proliferation [96]. Mitochondrial genome-derived (mt)-circRNAs were found to be highly expressed in the plasma exosomes of CLL patients. The endogenous reduction of mc-COX2 can affect mitochondrial functions, suppress cell proliferation, and induce cell apoptosis. The upregulation of mc-COX2 was positively associated with leukemogenesis and worsening survival of CLL patients. Our findings prove that mc-COX2, a critical mt-circRNA highly expressed in plasma, derived from CLL cells and delivered by exosomes, is associated with the progression and prognosis of CLL [97]. CircRASSF2 was up-regulated in serum exosomes from PTC patients. The study demonstrates that circRASSF2 modulates PTC progression through the miR-1178/TLR4 pathway, which indicate that circRASSF2 may serve as a promising therapeutic target for the treatment of PTC patients [98]. In terms of clinical diagnostic biomarkers, studies have demonstrated the profiles of differentially-expressed circRNAs in extracellular vesicles in serum of patients with thyroid papillary carcinoma [105] and endometrial carcinoma [106]. These results suggest that circRNAs derived from EV has diagnostic potential.

In summary, a large number of studies have shown the great potential of exosomes associated circRNAs as potential biomarkers in liquid biopsies, as illustrated in the Table 2. We will further explore the mechanisms involved in the disease process in the future.

**Table 2:** Dysregulation of exosomal circRNAs in other diseases

circRNA	Diseases	Expression	Biological function	Mechanism	Citation
CircHIPK3	Cardiomyocytes	Upregulated	Decrease in oxidative stress-induced CMVECs dysfunction	CircHIPK3/miR-29a/IGF-1	[107]
cZFP609	Ischemic diseases	Upregulated	Inhibit endothelial angiogenic activity induced by hypoxia	cZFP609 /HIF1α/VEGFA	[108]
circHIPK3	Ischemic injury of the skeletal muscle	Downregulated	Improve blood perfusion, inhibit ischemia induced pyroptosis	circHIPK3/miR-421 /FOXO3a	[32]
circ-Rtn4	Osteoporosis	Upregulated	Increase cell activity and inhibit apoptosis	circ-Rtn4/miR-146a/TNF-α	[109]
circRNA-0077930	Vascular smooth muscle aging	Upregulated	Promote cellular senescence of VSMCs	circRNA-0077930/miR-622/Kras	[110]

*CMVECs* cardiac microvascular endothelial cells

## Exosomal circRNAs in other disease

Not only do exosomal circRNAs play a vital role in tumors, but also play an important role in diseases other than tumors, such as nervous system diseases and cardiovascular diseases, as summarized in Table 3.

**Table 3:** Exosomal circRNAs in clinical applications

CircRNA	Disease type	Specimen source	Specimen type	Expression	AUC	Citation
hsacirc_020135	PTC	PTC/benign thyroid goiter	Serum	Downregulated	–	[105]
hsa circ 0002577	Endometrial cancer	Endometrial cancer/healthy	Serum	Upregulated	–	[106]
hsa_circ_0065149	GC	GC/healthy/gastritis	Plasma	Downregulated	0.769	[100]
three-circRNA signature	HCC	HCC/healthy	Serum	Upregulated	0.89	[99]
hsa-circRNA-0088088	Breast cancer	Breast cancer/healthy	Serum	Upregulated	–	[102]
hsa-circRNA-00005795	Breast cancer	Breast cancer/healthy	Serum	Downregulated	–	[102]
CircRNA-002178	LUAD	LUAD/healthy	Serum	Upregulated	0.9956	[104]
hsa_circRNA_0056616	LUAD	With lymph node metastasis/without	Plasma	Upregulated	0.812	[103]
Circ-0051443	HCC	HCC/healthy	Plasma	Downregulated	0.8089	[80]
circ-0004771	CRC	CRC/healthy	Serum	Upregulated	0.88	[114]
hsa_circ_0087862	Immune-mediated demyelinating	Immune-mediated demyelinating/healthy	Cerebrospinal fluid	Upregulated	1	[115]
circ-KIAA1244	GC	GC/healthy	Plasma	Downregulated	0.7481	[116]

*PTC* papillary thyroid carcinoma, *GC *gastric cancer, *HCC *hepatocellular carcinoma, *LUAD* lung adenocarcinoma

Human platelets are particularly rich in circRNA compared to other hematopoietic cell types, and since platelets are essential for central physiological processes such as hemostasis, wound healing, inflammation, and cancer metastasis, these findings could greatly expand the potential of circRNA as a prognostic and diagnostic biomarker [27, 111, 112]. Exosomes from the extracellular space of the brain, where differentially-expressed circRNAs might be related to the growth and repair of neurons, the development of the nervous system, and the transmission of nerve signals. The most highly correlated pathways that identified were involved primarily with glutamatergic synapse and the cyclic guanosine monophosphate-protein kinase G signaling pathway [113]. CircHIPK3 in hypoxic exosomes (HPC-exos) plays a role in cardiac microvascular endothelial cells (CMVECs) under oxidative conditions through miR-29a-mediated IGF-1 expression, leading to a decrease in oxidative stress-induced CMVECs dysfunction. These data suggest that the exosomal circRNA in CMs is a potential target to control CMVECs dysfunction under oxidative conditions [107].

To explored the role of smooth muscle SIRT1 in endothelial angiogenesis after ischemia and the underlying mechanisms, we performed a femoral artery ligation model using VSMC specific human SIRT1 transgenic (−Tg) and knockout (KO) mice. −Tg VSMCs inhibited endothelial angiogenic activity induced by hypoxia via the exosome cZFP609. The cZFP609 was delivered into ECs, and detained HIF1α in the cytoplasm via its interaction with HIF1α, thereby inhibiting VEGFA expression and endothelial angiogenic functions [108].

## Conclusion

As an important carrier of exosomes, circRNAs play an important regulatory role in various physiological and pathological biological processes, such as inflammation, tissue repair, cell proliferation, migration, invasion, metastasis, apoptosis, and chemotherapy & radiotherapy resistance. Due to their unique features and high specificity, their ability to mediate cell communication, what is more, exosomal circRNAs is abundant, stable, and present in different extracellular fluids, including saliva, blood, and urine [28], making it an ideal diagnostic marker and therapeutic target as well as potential vectors for drug delivery. However, compared with other ncRNAs, such as miRNA and lncRNA, the studies of circRNAs in exosomes are still incomplete. Therefore, we need to further explore its functional mechanism.

At present, the study of the structure of endogenous circRNAs sponges may contribute to the design and development of effective artificial sponges to regulate disease progression. As a stable and effective miRNA inhibitor, the artificial miRNA "sponge" technique may be a new strategy for RNA gene therapy in the future. It can simultaneously inhibit the expression of other miRNAs and produce a more long-lasting inhibiting effect. Therefore, the use of nanoparticles to deliver circRNAs to generate specific types of exosomes provides an option for selective targeted cancer cell elimination [117–120].

However, the specificity of exosomal circRNAs structure and the insufficiency of technology limit the discussion of its function. There are still many challenges and difficulties in clinical application. First, a standardized method for collecting, processing and isolating exosome samples has not yet been established [28, 121]. Ultracentrifugation method is the gold standard for exosome separation, but it is time and labor-consuming, requires a large amount of raw materials and expensive equipment, and is inefficient for high-throughput measurement [122], which cannot be effectively applied to clinical work. Second, in the clinical collection of patient samples, blood or other body fluids contain many impurities, such as protein complexes, nucleic acid lysates, etc., which are challenging to extract correct exosomes. Third, circRNA and its corresponding linear mRNA sequences are partially overlapped, so circRNA expression and function cannot be accurately assessed. Fourth, due to the low abundance of circRNAs, it is difficult to detect them in exosomes using accurate methods. Therefore, it is important to develop and use appropriate methods and techniques to elucidate the molecular mechanisms and regulatory networks of exosomal circRNAs.

## Abbreviations

AFM: Atomic force microscope
CD: Cluster of differentiation
CircRNAs: Circular RNAs
COX: Cyclooxygenase
EM: Electron microscope
ESCRT: Endosoma-sorting complex required for transport
EV: Extracellular vesicle
MHC: Major histocompatibility complex
MMP: Matrix metalloprotease
HSP: Heat shock protein
HUVECS: Human umbilical vein endothelial cells
lncRNA: Long non-coding RNA
miRNA/miR: Micro-RNA
mRNA: Messenger RNA
MVB: Multi-vesicular body
TEM: Transmission electron microscopy
